# 

**Published:** 1890-01-25

**Authors:** 


### NOTICE TO CORRESPONDENTS.

All MS., Letters, Books for Review, and other matters intended for the Editor, should be addressed THE EDITOR, THE LODGE, PORCHESTER SQUARE, LONDON, W.

### Notice to Advertisers and Subscribers.

All Advertisements, Orders for Copies of THE HOSPITAL, and Business Communications should be addressed to The Publisher, *not to the Editor*, THE HOSPITAL, 139-140, Salisbury-court, Fleet-street, E.C.

### Bound Volumes of "The Hospital."

Vol. VI., containing full page portraits of T.R.H. the Prince and Princess of Wales, and Miss Florence Nightingale, is now ready, 4s., post free. Cases for binding, may be had of the Publisher, at 1s. 6d. each.

### To Residents, Secretaries, and Matrons

The Editor will be glad to have the Regulations and each Report as issued by Asylums, Hospitals, Convalescent and Nursing Institutions, as well as those of other Charities, and an early copy in duplicate of all Appeals and Festival Notices, etc., addressed to *The Editor, The Lodge, Porchester-square, London, W.* Any superintendent, secretary, matron, or other chief official who regularly sends such information may be registered, on application to the Publisher, to receive free of cost a cover for binding THE HOSPITAL in half-yearly volumes.

### Scraps and Gleanings.

Dr. Downe has been seriously ill with influenza.

Dr. Dundas Grant was married last week to Miss Nellie Frith.

The Cyprus Society will hold a drawing-room meeting on February 15.

A successful concert in aid of the Dublin Dental Hospital has been held.

The Geneva Red Cross Society will erect a memorial to the late Empress Augusta.

The Countess of Zetland unveiled the bust of Dr. John Denham, of Dublin, last week.

Surgeon Parke, M.D., has been appointed by the Khedive to the 4th class of the Medjidieh.

Bath Eastern Dispensary issues a good report. They have a balance in hand of £50.

Chicago is to have an infectious hospital, and a special ward for diphtheria, built by a lady.

Dwellers at Kew lose 300 hours of sunlight yearly as compared with dwellers on the Isle of Wight.

St. Petersburg citizens have undertaken to educate the four sons of the late Professor of Hygiene.

Boston Hospital has paid off the debt of £200 on the building fund, and starts 1890 free from debt.

Edinburgh medical students are being instructed in sick-room cookery by a charming young lady cook.

Prince Albert Victor has opened the Women's Hospital at Lucknow, in connection with Lady Dufferin's Fund.

Barrington's Hospital treated 344 in-patients and nearly 3,000 out-patients last year. Total expenditure £1,141.

The Duchess of Fife has become patroness of the Scottish National Society for the Prevention of Cruelty to Children.

On January 8th three persons were bitten at Leicester by a mad dog. They have all been to M. Pasteur to be inoculated.

Hull Sunday and day school collections for the infirmary reached £220. Our congratulations to the generous scholars.

The Campbell bequest of £200,000 is to be expended on a college for Belfast; any surplus to be devoted to founding a hospital.

The asylum at Worcester, Massachusetts, has been destroyed by fire. The patients were all rescued, but there were some harrowing scenes.

At the annual meeting of the committee of the Civil Service Lifeboat Fund it was reported that the number of subscribers had reached 10,000.

A gold medal has been presented to a lady Russian doctor by the Society of Friends of Research for her essay on "Female Population in the Caucasus."

Derbyshire General Infirmary will, in future, admit physicians and surgeons to its staff who have graduated at any university in the United Kingdom.

The Marquis of Lorne has promised to preside at the annual festival of the Royal Blind Pension Society, which is to be held in May at the Hotel Metropole.

Influenza is still claiming many victims. The increase of deaths chronicled daily in the *Times* is proof enough that the epidemic is far more serious than most people admit.

Stockton Hospital ladies' committee, on the proposal of the Marchioness of Londonderry, have decided that the visiting lady for the month shall provide books and papers for the patients.

A committee has been appointed, of which Dr. A. B. Duffin is treasurer, to present Mr. Wood with a testimonial in recognition of his long and valued services as an anatomical and surgical teacher at King's College.

The Borough Council is still quarrelling at Huddersfield over the site for the proposed fever hospital. The language used by Alderman Glendinning at the meeting on January 15th was a disgrace to the Council.

At an inquest held at Beckenham the coroner arrived late, and, having kept the jury waiting for a considerable time, he promptly fined himself the sum of one guinea, which was forwarded as a contribution to the funds of the local hospital.

The Queen has forwarded gifts of pheasants to the London Hospital and to the Hospital for Women, Soho. Her Majesty has also sent a large quantity of cast linen for use in King's College Hospital, St. George's Hospital, and the Chelsea Hospital for Women.

Miss Dhunbai Sorabjee Mervanjee Master has been declared by the Principal of the Grant Medical College, Bombay, duly qualified as a medical practitioner, after a special examination held for her, and granted a diploma. This makes five Parsee lady graduates of the Medical College.

An International and Industrial Exhibition will be opened at Leeds in May. In addition to the ordinary features of exhibitions, a special section will be devoted to the housing of the poor, and many prizes and medals will be offered for plans, models of houses and apartments, and specimens of sanitary and other fittings.

The council of the South London Fine Art Gallery have received, in response to an appeal signed by Sir F. Leighton, an offer from a friend to build a handsome art gallery as a gift to South London. The committee now require only enough money to purchase the site, etc.—some £4,000—of which they have already received £1,100.

In consequence of the great increase in the number of patients applying for treatment in the out-patient department of Chelsea Hospital for Women, Fulham-road, it has been decided by the Board of Management to open that department every week day, instead of as heretofore on four days only. The new days will be Thursdays and Saturdays, the hours of attendance being from 1.30 p.m. to 3 p.m.

